# Teenagers’ understandings of and attitudes towards vaccines and vaccine-preventable diseases: A qualitative study^☆^

**DOI:** 10.1016/j.vaccine.2013.04.023

**Published:** 2013-05-24

**Authors:** S. Hilton, C. Patterson, E. Smith, H. Bedford, K. Hunt

**Affiliations:** a MRC/CSO Social and Public Health Sciences Unit, 4 Lilybank Gardens, Glasgow G12 8RZ, United Kingdom; b Centre for Epidemiology and Biostatistics, UCL Institute of Child Health, 30 Guildford Street, London WC1N 1EH, United Kingdom

**Keywords:** Teenagers, Adolescents, Disease, Risk, Vaccines, Immunisation, Understanding

## Abstract

**Background:**

To examine immunisation information needs of teenagers we explored understandings of vaccination and vaccine-preventable diseases, attitudes towards immunisation and experiences of immunisation. Diseases discussed included nine for which vaccines are currently offered in the UK (human papillomavirus, meningitis, tetanus, diphtheria, polio, whooping cough, measles, mumps and rubella), and two not currently included in the routine UK schedule (hepatitis B and chickenpox).

**Methods:**

Twelve focus groups conducted between November 2010 and March 2011 with 59 teenagers (29 girls and 30 boys) living in various parts of Scotland.

**Results:**

Teenagers exhibited limited knowledge and experience of the diseases, excluding chickenpox. Measles, mumps and rubella were perceived as severe forms of chickenpox-like illness, and rubella was not associated with foetal damage. Boys commonly believed that human papillomavirus only affects girls, and both genders exhibited confusion about its relationship with cancer. Participants considered two key factors when assessing the threat of diseases: their prevalence in the UK, and their potential to cause fatal or long-term harm. Meningitis was seen as a threat, but primarily to babies. Participants explained their limited knowledge as a result of mass immunisation making once-common diseases rare in the UK, and acknowledged immunisation's role in reducing disease prevalence.

**Conclusions:**

While it is welcome that fewer teenagers have experienced vaccine-preventable diseases, this presents public health advocates with the challenge of communicating benefits of immunisation when advantages are less visible. The findings are timely in view of the Joint Committee on Vaccination and Immunisation's recommendation that a booster of meningitis C vaccine should be offered to teenagers; that teenagers did not perceive meningitis C as a significant threat should be a key concern of promotional information. While teenagers’ experiences of immunisation in school were not always positive, they seemed enthusiastic at the prospect of introducing more vaccines for their age group.

## Highlights

• Explored understandings of diseases and knowledge and experiences of vaccination.
• Teenagers had limited knowledge and little direct experience of the diseases.
• Participants attitudes towards receiving vaccines’ varied.
• The success of mass immunisation programmes is associated with disease perceptions.
• Need to engage with teenagers to address misconceptions about vaccines and diseases.

## 1 Introduction

Immunisation schedules are amended as new vaccines become available and scientific knowledge increases [1]. Traditionally, few vaccines have been offered to teenagers in most countries. For decades in the UK, tetanus and polio were the only vaccines offered to 13-18 year olds to boost immunity to these infections throughout adulthood; low dose diphtheria was added to this school-leavers’ booster in 1994 [2]. In Wales, the only UK country to publish coverage of the teenage booster, uptake is significantly lower than vaccines given in early childhood (33.6% compared with 95%+) [3]. However, human papillomavirus (HPV) vaccine introduced in 2008 for 12-13 year old girls achieved almost 90% uptake in England [4] indicating that immunisation of this age group is not only feasible, but can be highly successful. It has been suggested that other vaccines should be included in the teenage vaccine programme, for example booster doses of pertussis vaccine [1,5,6]. In January 2012 the Joint Committee on Vaccination and Immunisation (JVCI) advised that a dose of Meningitis C vaccine be moved from the infant to the teenage immunisation schedule [7]. In some areas, MMR vaccine has been offered to teenagers who missed earlier doses due to unsubstantiated fears over its safety, a major concern in the 2000s, at the same time as the teenage booster [8,9]. The introduction of varicella and of hepatitis B vaccines have been under consideration for some time by JCVI, with administration in the teenage years a possible option [5,6].

Due to the success of immunisation, most teenagers have little experience of vaccine-preventable diseases. This inexperience may both increase the challenge of maintaining high coverage and focus attention on alleged adverse effects of immunisation [10]. Attitudes to diseases and vaccines are important predictors of immunisation uptake [11–13]. People's vaccine decisions balance ‘complex and sometimes contradictory’ concerns [14], including perceptions of the risks and severity of diseases and the risks of vaccines [10]. Although decision-making is routine for some [15,16], assessments of risks or uncertainties associated with vaccines or diseases [17] often emerge after discussion with others [18], including health professionals [10,16,19]. Understanding people's knowledge of, and attitudes towards, vaccines and diseases is important in understanding vaccine uptake.

A wealth of research has focused on parents’ attitudes towards childhood vaccinations [11,13,19–23], and some examining parents’ knowledge and understanding of vaccine-preventable diseases. In these studies meningitis, diphtheria, tetanus and poliomyelitis are commonly perceived as severe, but rare [13,14,24–27]. Perceptions of the severity of measles are inconsistent, with some studies reporting it to be considered severe [13,14,27–29] and others mild [10,30]. Similarly, mumps and rubella are variously perceived as severe [14,29] or mild [24,26,27]. These contradictory findings may arise from different methodological approaches, but there is broad agreement that parental understanding of diseases is limited.

The few published studies investigating teenagers’ understanding of vaccines and vaccine-preventable diseases have focused on HPV. These have found a general lack of awareness of the virus [31], in particular its relationship with cervical cancer, even in young women who were the target of the high-profile educational campaign accompanying the vaccine's introduction [25]. Hepatitis B is also little understood by teenagers [32], even in countries where the vaccine is part of the routine infant schedule [33].

To our knowledge this is the first UK study offering in-depth accounts from both young men and women on a range of vaccine-preventable diseases and their attitudes to immunisation. We set out to explore teenagers’ understandings, beliefs and experiences of nine diseases routinely vaccinated against (HPV, meningitis, tetanus, diphtheria, polio, whooping cough, measles, mumps and rubella) and two vaccine-preventable diseases that, it has been suggested, should be added to the UK's teenage immunisation programme (hepatitis B and chickenpox). We also examined views about vaccination more generally, exploring personal experiences of vaccination and negotiating consent, and views about whether more vaccines would be acceptable. With an expanding adolescent vaccination programme, it is important to establish teenagers’ attitudes and knowledge to guide information campaigns.

## 2 Method

### 2.1 Sampling and recruitment

Twelve focus groups were conducted between November 2010 and March 2011 with teenagers aged between 13 and 18 living in Scotland. Purposive sampling was used to recruit a sample of diverse socio-economic background, geographical location, age and sex (see Table 1). Teenagers were recruited through posters, leaflets and advertisements placed in settings including schools, community facilities and sport facilities. Advertisements inviting interested parties to contact the researcher (ES) were placed on websites such as Facebook and Bebo. To allow recruitment from a broad range of socio-economic backgrounds, key community leaders in socially deprived and advantaged areas were approached to help identify community groups. Groups predominantly consisted of friendship groups. Each participant was given a £10 shopping voucher to cover expenses and thank them for contributing.

### 2.2 Data collection and analysis

Focus groups were facilitated by ES and carried out in local community facilities. Discussions lasted between 45 and 80 min, covering issues relating to participants’ understandings of diseases and vaccines. In each group the researcher first asked participants about their vaccination histories since childhood, then prompted them to describe their understandings of specific diseases and their symptoms, and finally prompted them to discuss their experiences of, and attitudes towards, vaccinations. With participants’ permission, discussions were audio-recorded and transcribed verbatim. Each transcript was checked and imported into NVivo 8 to enable systematic comparisons to be made across the large amount of data. Data were thematically coded and systemically charted following framework analysis principles [34], allowing data to be rigorously examined and cross-compared to identify common reasoning and themes, and ideas that are less common or are specific to certain subgroups or individuals. Themes were coded by ES and CP and checked by SH. Final analysis of frameworks was conducted by CP and SH. During analysis, attention was paid to deviant or contradictory cases [35] and to group dynamics, using full transcripts supplemented by field-note observations [36].

### 2.3 Reporting data

To report data we selected concisely-expressed quotations that typify responses around key themes, and some discussion extracts that convey the types of group interactions that occurred. Focus group methods can generate dynamic data by encouraging discussion between group members [37]. Chaotic conversations in more animated groups can make individual speakers difficult to identify; field notes taken during the discussions facilitated identification of speakers. Participants have been assigned pseudonyms to contextualise contributions while preserving anonymity.

Ethics approval was obtained from the research ethics committee of the University of Glasgow's Law, Business and Social Sciences Faculty.

## 3 Results

Fifty-nine teenagers (29 girls and 30 boys) aged between 13 and 18 years old took part in 12 focus group discussions (Table 1). Participants were asked about their immunisation history. Forty-six had been vaccinated as teenagers with HPV, Td/IPV or a travel vaccine; 13 had received no vaccinations. Of those 13, four had not been offered vaccinations as teenagers, six had accidentally missed vaccines and three had decided, with their parents, to refuse vaccines. Two participants had never received any vaccinations due to their parents’ beliefs about immunisation.

With the exception of chickenpox, participants typically had little or no direct experience of the diseases discussed, except for one who recalled contracting mumps (Lizzie, FG4), and another whooping cough (Olivia, FG6). Most participants’ views about diseases therefore derived from indirect experiences. Participants were generally uninquisitive about vaccine-preventable diseases, with only two describing actively seeking information, from the Internet (Olivia, FG6) and their doctor (Tracy, FG6).

### 3.1 Perceptions of diseases

When assessing how threatening a disease is, participants tended to talk about two key factors: its prevalence in the UK and whether it could be fatal or cause long-term, debilitating and irreversible damage. Perceptions of risk were typically mediated by two factors: geographical location, and contemporaneousness. Participants frequently deemed diseases to represent threats in less developed countries, but not in the UK; one girl observed: ‘can you not just die from all of them if you were in like living in Africa or somewhere like that?’ (Daniele, FG9). Similarly, participants frequently perceived diseases as historical threats that are no longer relevant. As one girl explained, ‘It's been more like things that are more serious like in the past and now … people are more protected against them’ (Rebecca, FG8). Diseases associated with less developed countries or the past included tetanus, diphtheria, poliomyelitis, whooping cough, and tuberculosis. Participants described these as from ‘the past’, ‘the war’ (Murray, FG8) and ‘Victorian times’ (Steven FG9). One associated poliomyelitis with the ‘1940s’, corresponding with the outbreak of that decade. Explanations for reduced threat in contemporary Britain included successful mass immunisation programmes, advances in medicine and improvements in living conditions. Participants believed those diseases could be threatening again if immunisation was to cease. Participants’ awareness and understandings of each disease discussed are detailed in Table 2.

### 3.2 Understandings and concerns about immunisation

Advantages and disadvantages of immunisation were discussed. Participants expressed varied views about whether immunisation is a positive or negative intervention. Just over half of the participants presented positive images of vaccines protecting the population against disease. Wendy (FG12) stated: “It has been proved to, like, get rid of, or practically get rid of some diseases, like, in the population – so that's a positive thing”. Most participants displayed high trust in vaccines and little concern about side-effects. Participants discussing side-effects tended to focus on short-term rather than long-term effects, typically discomfort surrounding the administration of vaccines. Long-term side effects mentioned included fertility problems (Rebecca FG8; Laura FG7), negative effects on the immune system (Murray, Alan FG8) and reduced resistance to diseases being vaccinated against (Quentin FG7). Conversations about vaccinations were dominated by images of needles and the fear and pain associated with receiving injections. When elaborating on thoughts about needles and injections, participants often used negative language, describing them as ‘painful’ (Lewis FG8), ‘scary’ (Alison FG4), ‘sharp’ (Laura FG7) and ‘dagger-like’ (Rebecca FG8). Although many participants described their anxieties about injections at length, three groups discussed the notion that the unpleasantness of vaccinations tends to be exaggerated (see Box 1).

Some participants were extremely worried by vaccinations. One participant, who also explained that she did not feel a responsibility to be immunised because she had no dependents, stated: ‘I’d rather die in my sleep than have a jag’ (Samantha, FG4). Another did not perceive the benefits as outweighing the costs, stating: ‘I’d rather have cancer than get a very painful, numb arm I’ll actually kill my mum if she makes me get another one’ (Sula, FG5).

Participants were asked about receiving newly-introduced vaccines and combination vaccines. Discussions about combined vaccines tended to conclude that they are preferable as they reduce the overall number of injections needed, as illustrated in Kenny's (FG3) comment that “…the fewer, the better as far as I’m concerned”. Most groups responded positively to the suggestion of new vaccines being introduced to the school immunisation programme. Focus Group 9 typified these discussions:

**Chris:** I’d be delighted.
**Harry:** The more the merrier.
**Gayle:** It's going to help you, like if it's going to help you, you might as well get it.
**Chris:** It's for your good.
**Daniele:** I think any jag would be good to get.
**Darren:** Unless it's one of those massive needles (laughs)

Some were wary of new vaccines. One stated: “…they may have unknown side-effects if they’ve not been tried on people…like you don’t know what could happen in the long-term (Rebecca, FG8). Participants mentioned that they did not like being ‘used as guinea pigs’ (Veronica, FG11; Karl FG5; Amy FG3). While most participants were prepared to accept new vaccines regardless of the disease targeted, others considered whether specific diseases merited vaccination (see Box 2).

### 3.3 Beliefs about choice and responsibility

Participants were encouraged to discuss issues of choice and responsibility related to vaccination. Two positions were commonly presented: choice about vaccinations is desirable, and universal immunisation is advantageous. Some regarded choice as essential, while acknowledging the utility of vaccinations: “Refusing vaccinations is just silly. … But if there was a reason why a mother didn’t want their child to have that vaccination, they shouldn’t be forced to’ (Moira, FG3).” Steven (FG9) suggested: ‘it is good that it's voluntary because like you’re not actually forced into it … you can actually make your own decision.’ A participant who had opted out of vaccination against HPV felt that those who opt out can experience pressure due to social expectations: ‘Some people might still feel like because the majority maybe do get it, they might still feel pressured or feel like, you know, why are you not? … There's still quite an expectation for you just to get it.’ (Rebecca, FG8). It was suggested that promoting vaccinations as a social responsibility may make those who opt out feel guilty. Some described those who opt out of as “lazy” (Chris FG9), “stupid” (Daniele FG9) and “selfish” (Wendy FG12). However, the same participants recognised that there were legitimate reasons to opt out on medical or religious grounds. Some respondents believed compulsory vaccinations could generate opposition to vaccine acceptance in reaction to the lack of choice. Participants in most groups recognised vaccination as beneficial to society as well as the individual. Eric (FG1) believed that: “…people have a responsibility to receive vaccines if they want to keep themselves safe and not pass it on to the others”. There was little acknowledgement that some people cannot receive vaccines.

### 3.4 Experiences of vaccination and decision-making

Not all participants’ experiences of receiving vaccines were negative (Box 1). Participants in one group discussed an unexpectedly straightforward vaccination experience in which the immuniser engaged the student in conversation while performing the injection, which made the process more pleasant than anticipated.

Participants discussed circumstances of vaccinations administered in school. Participants in Focus Group 1 expressed concerns about privacy, personal space and the potential discomfort of being watched by peers during vaccination. Students described a fellow pupil who had to remove her shirt to receive a vaccine, which was agreed to be an undesirable experience. While some participants disliked being watched, others described witnessing vaccination in others as similarly distressing: “It's not good because you can see everyone else getting theirs done, and you’re thinking, ‘oh, that's what's going to happen to me’ and you start to freak out” (Deborah, FG12).

Some responsibility for generating anxiety was attributed to those administrating the vaccines. One participant stated: “my biggest worry is just knowing that I’m in safe hands as I don’t know them from Adam” (Kieron, FG6). Another complained that: “you’re getting a jag and they’re telling you about more jags, it's ‘I don’t want to hear about more jags”’ (Samantha, FG4). Some with particular anxieties had opted to receive vaccines at their GP surgery, rather than in school, although they also acknowledged it would be impractical to deliver the whole programme in that way (Ailsa FG 2). Mild side-effects of vaccination were described. For most participants anxiety was the most significant concern, causing them to feel sick, faint, tearful or unable to concentrate on schoolwork (Brenda, FG2, Deborah FG12, Sammy, FG4).

Participants were encouraged to discuss the process of deciding whether to receive vaccinations or not. Some discussed vaccinations with their parents and felt able to influence the decisions: “mum says it was up to me if I wanted it or not” (Eric, FG1). However, participants more commonly felt that the decision was ultimately taken by their parents, primarily mothers. Participants typically acknowledged they could contribute to the process, but many viewed it as a false choice, in which the only legitimate option was to agree. Anna (FG1) typified this, explaining: “my mum will probably just say ‘do you want it?’ and I’ll be like ‘not really’ but she’ll just sign it (consent form) anyway no matter what”.

## 4 Discussion

To our knowledge this is the first UK study investigating in-depth the understandings, attitudes and experiences of teenage boys and girls regarding a range of vaccines and vaccine-preventable diseases. The broad finding that teenagers’ understandings of vaccine-preventable diseases are limited is in line with previous research [25,27,38–41]. This study identifies many of the same gaps in understanding as previous research with parents of young children [27], supporting the conclusion that understanding of diseases is closely related to experience of diseases. This is illustrated by the finding that chickenpox was both the most commonly experienced and widely understood disease discussed. This supports the suggestion that successful immunisation has reduced knowledge of diseases, their severity and possible long-term consequences [10,42].

Teenagers often referred to chickenpox when describing other diseases about which they knew little. For example they imagined that measles, mumps and rubella might resemble severe cases of chickenpox. Rubella was not associated with the risk of foetal damage, and participants were divided about the severity of both mumps and measles, echoing previous research [10,13,14,27,29]. Participants (particularly girls) were most aware of HPV infection but it was nonetheless subject to misunderstandings; boys commonly believed it to only affect girls, and both sexes demonstrated confusion about its relationship with cervical cancer, again echoing existing research [25,40,41,38,43]. As HPV infection is complex and fundamentally different from other vaccine-preventable diseases, so misunderstandings are not surprising. However, as the primary aim of HPV vaccination is the prevention of cervical cancer, this is somewhat disappointing. Many female participants had received the vaccine relatively recently, and should have been availed of this information. This emphasises the need to develop information that meets this young audience's specific needs.

In contrast to studies where teenagers demonstrated low awareness of hepatitis B [32,33,39,38], our participants displayed relatively good knowledge of its transmission, and perceived it as a threat. When assessing which diseases were most threatening, participants mentioned two key factors: its prevalence in the UK, and whether it could be fatal, debilitating, or cause long-term harm. In line with previous research [11,28], some of the participants’ vaccine decision-making seemed to be strongly influenced by perceptions of disease severity, emphasising the need for clear information. Many diseases were seen as historical, and no longer threats in the UK, complementing findings of research with parents [27]. While meningitis was considered one of the more life-threatening diseases, teenagers tended to think only babies were at heightened risk. Since teenagers are at greater risk than babies of a poor outcome from Meningococcal C infection [44], this is concerning. This misconception is particularly relevant as the JCVI have recommended a dose of Meningococcal C vaccine be offered to all teenagers, and information about it will need to feature prominently in accompanying information.

Overall the participants were very aware of vaccines’ impact on reducing diseases, and some were enthusiastic at the prospect of more becoming available. However, given the nature of recruitment to the study, our findings may not be representative of all adolescents, and may reflect the views of more enthusiastic or interested teenagers.

The teenage years are a key opportunity to promote vaccination to future parents. For example, some of the participants expressed concern that they may be ‘guinea pigs’ for new vaccines, echoing concerns found in studies of parental attitudes [45]. As such, improving teenagers’ understandings of how vaccines are developed, introduced and monitored could benefit vaccine-related decision making and uptake throughout life.

Our findings also revealed that although teenagers are encouraged to make decisions about immunisation, the decision is often made by parents, particularly mothers. Parents may view the process differently; in one study of 300 parents of 11-12 year olds most felt that decisions about vaccines should be made jointly with parents, but almost half agreed that a well-informed child should be able to request vaccines without parental consent [20]. The teenage years are an ideal time for parents to involve children in making health-related decisions, particularly since 16-18 year olds are legally entitled to consent to treatment, and under-16s who can demonstrate they fully understand the proposed intervention can also give consent (“Gillick” competent). It is unclear to what extent parents understand this, but guidance on consent has been specifically developed for them [46].

For many participants the administration of vaccines represented the greatest source of worry. This is important information for vaccine providers; efforts must be made to deliver school-based vaccination in ways which take teenagers concerns seriously and reduce anxiety and negative experiences. One study [3] reported that significantly higher uptake of teenage boosters has been achieved when administered in schools, compared with in general practice, and another [47] found that combining the delivery of HPV vaccine for girls with the teenage booster for girls and boys resulted in higher uptake of both, and a mutually-supportive atmosphere among recipients. This is reassuring given the forthcoming addition of Meningitis C vaccine to the adolescent schedule, which will require teenagers to receive more than one vaccine at time.

Although a total of 59 young people were included in this study, and particular effort was made to include teenagers from diverse socio-economic backgrounds and geographical locations in Scotland, no students from ethnic minority backgrounds took part, which may limit the generalisability of the findings to the wider UK population. Nevertheless, the findings give important insights into teenagers’ understandings, attitudes and experiences. In future research it will be useful to establish whether the belief that meningitis is most risky for babies is widely held. No clear differences in understandings and experiences based on socioeconomic status, age or sex emerged from the data.

## 5 Conclusions

These young people will be the next generation of parents. Offering additional vaccines to adolescents provides a real opportunity to build their confidence and knowledge about the value of vaccines, which may in turn positively influence their future decisions for their own children.

Our findings contribute to a body of literature highlighting a lack of public understanding of diseases that have become uncommon due to successful mass immunisation. This presents public health and immunisation promoters with the challenge of communicating the benefits of immunisation at a time when the advantages are less visible. While it is fortunate that teenagers have little personal experience of vaccine-preventable diseases, it contributes to the challenge of developing ways to engage with them to increase their understanding and awareness of the threats posed by vaccine-preventable diseases, so that they are accepting of vaccines for themselves and will be able to make informed choices about immunisation for their own children as the parents of tomorrow.

## Figures and Tables

**Table 1:** Characteristics of focus groups and participants.

Focus group	Recruited from	Pseudonym	Sex	Age
1	Mixed area: Highland region	Douglas	Male	14
	High school	Anna	Female	14
		Eric	Male	14
		Victor	Male	14

2	Mixed area: Highland region	Struan	Male	16
	High school	Ailsa	Female	16
		Sharon	Female	16
		Brenda	Female	16
		Vincent	Male	16
		Frank	Male	16

3	Mixed area: Highland region	Amy	Female	15
	High school	Moira	Female	15
		Kenny	Male	15
		Dean	Male	15
		Diana	Female	15
		Naomi	Female	15

4	Deprived area: West Lothian	Alison	Female	13
	High school	Lizzie	Female	14
		Vicky	Female	13
		Samantha	Female	14

5	Deprived area: West Lothian	Angus	Male	14
	High school	Sula	Female	13
		Tina	Female	14
		Karl	Male	15

6	Mixed area: Edinburgh	Chloe	Female	17
	Youth Employment initiative	Olivia	Female	16
		Stuart	Male	17
		Kieron	Male	17
		Tracy	Female	17
		Grace	Female	16

7	Deprived area: Glasgow, East End	Stacy	Female	14
	Youth group	Quentin	Male	18
		Euan	Male	17
		Laura	Female	16
		Finn	Male	17
		David	Male	15
		Tony	Male	14
		Neil	Male	16
		Darren	Male	15

8	Affluent: Edinburgh	Rebecca	Female	17
	Youth group	Murray	Male	14
		Gary	Male	13
		Alan	Male	15

9	Mixed: Renfrewshire	Chris	Male	15
	Youth group	Harry	Male	15
		Darren	Male	15
		Steven	Male	15
		Gayle	Female	15
		Daniele	Female	15

10	Deprived: North Glasgow	Lewis	Male	15
	Education support facility	Edward	Male	15

11	Mixed: Renfrewshire	Struan	Male	15
	Youth group	Rhea	Female	15
		Trisha	Female	15
		Veronica	Female	15

12	Affluent: Glasgow, West End	Christine	Female	17
	Youth group	Deborah	Female	17
		Wendy	Female	15

**Table 2:** Participants awareness and understandings of vaccine-preventable diseases.

Disease	Awareness of disease	Understanding of disease process and symptoms
Diseases viewed as most threatening:		
Meningitis	Most had heard of meningitis, and perceived it as severe and quite prevalent. Not typically seen as a particular risk to teenagers; babies believed to be at greatest risk, due being the focus of a Men C public information campaign.	Almost half of participants described symptoms they associated with meningitis, including: skin lumps and rashes; throat problems; muscle pain; and blindness. Participants mentioned a television campaign promoting the “tumbler test” to identify whether a rash is suggestive of meningococcal septicaemia.

Hepatitis B	Participants had typically heard of hepatitis B but claimed no knowledge of it. They regarded it as causing debilitating, long-term damage. Commonly associated with drug abusers.	Participants offered relatively accurate descriptions of transmission when prompted, associating it with sexual transmission and infected blood from ‘sharing dirty needles’ and ‘bad tattoos’ (Stuart, FG6). Participants were reluctant to guess symptoms.

HPV	Girls typically demonstrated some awareness of HPV and many had recently been vaccinated against it. Boys demonstrated relatively little awareness. Participants typically perceived it as a prevalent infection, and many viewed it as a serious disease.	Boys commonly believed the virus only affects females. Some girls expressed similar beliefs: ‘I don’t think [boys] can catch HPV very easily’ (Tina, FG5). Doubts that HPV can infect males coexisted paradoxically with widespread recognition that it is transmitted sexually. Some demonstrated confusion about the association between HPV and cancer (see Box 3).

Diseases of uncertain threat:		
Measles	Typically believed to be in circulation within the UK, but unlikely to be contracted. Participants evenly divided between those who believed measles can cause long-term damage and those who believed it to be mild.	Many offered accurate descriptions of symptoms: itchy red spots and rashes; swelling; and fever. Commonly confused with chickenpox; some suggested it may be a more severe form (see Box 4).

Mumps	Participants unsure whether mumps is in circulation in the UK. Some believed it to be extremely prevalent. One girl described mumps as an ‘old fashioned disease’ (Trisha, FG11) Participants evenly divided between those who believed mumps to be severe and those who did not.	Mumps was commonly correctly associated with glandular swelling. Some incorrectly suggested that it causes chickenpox-like rashes on the skin. One boy believed that mumps is primarily contracted by males.

Rubella	Few had heard of rubella or German measles.	Those who attempted to describe symptoms mentioned irritating spots, a chickenpox-like rash, muscular problems and swelling of the face and neck. Some believed it may be ‘like a severe case of chickenpox’ (Chloe FG6, Darren FG9, Christine FG12). No participants described the mode of transmission or acknowledged the risk of foetal damage.

Diseases viewed as least threatening:		
Tetanus	Commonly viewed as a historical threat.	Few participants claimed to know the symptoms of tetanus. Two correctly associated it with lockjaw (Murray FG8, Douglas FG1). Participants accurately associated tetanus with wounds coming into contact with bacteria, but few knew the environmental conditions in which the organism lives. Suggested locations included rust and rusty metal, used needles, broken glass, and animal bites. Two correctly associated it with soil, but incorrectly linked it to rust.

Diphtheria	Most had heard of diphtheria. Typically perceived as an historical disease, but many were aware that they had been, or would in future be, vaccinated against it, and so wondered if it might still be in circulation.	None could describe its symptoms, and participants were particularly reticent to offer descriptions of its transmission.

Poliomyelitis	Most had heard of poliomyelitis, typically referring to it as ‘polio’. Awareness associated with having recently been vaccinated against it. Widely viewed as a historical threat, and associated with less developed countries.	It was generally associated with leg-related skeletal problems, including being wheelchair-bound and one leg being longer than the other. No participant mentioned respiratory muscle paralysis. Few could describe its transmission.

Whooping cough	Fewer than half were aware of being vaccinated against whooping cough. Generally not considered to be a threat to people living in the UK.	Commonly described as a severe cough and sometimes associated with coughing up blood or ‘black mucus’. No participants recognised that it can be fatal.

Chickenpox	Generally seen as a routine and trivial part of childhood, only problematic if contracted in adulthood. Participants who had not yet experienced chickenpox expressed anxiety about contracting it in future: ‘I’ve never had it. I don’t want to get it when I’m older, you can die.’ (Chris, FG9).	Participants tended to describe signs and symptoms of chickenpox accurately. It was seen as very contagious and mild, and associated with itchy red spots on the skin that can leave pock marks.

## Box 1 The discomfort caused by vaccination has been exaggerated

Sharon: Until I had that last jag, the HPV I had, I imagined them (the needle) being quite a big thing…
Ailsa: Yeah so did I.
Sharon: …but actually, now, like I think before that, like, any jags I’d had, when I was young, I didn’t really understand what was going on. But since I’m kind of… I’m older and I kind of saw that one, then I realised that the needle's actually quite small and they’re not as big as I’d imagined they were.
Facilitator: **Yeah**.
Ailsa: I found it helped a lot. Actually, I found with the HPV ones as well, I mean, when I got my flu one done, I didn’t actually care all that much. Ok, it stung a wee bit, but it wasn’t that bad. I mean, it was just kind of like, it felt like a wee tiny paper cut and that was it. But I mean, I can actually just sit there and watch the needle going in, but without any problem or anything whereas, when I was younger, I would have been screaming.

*Group 2*

## Box 2 The usefulness of a vaccine depends on the disease it prevents

Kenny: Chickenpox vaccine wouldn’t really benefit most people…
Diana: I would.
Kenny: … ‘cos most people have had the disease already.
Diana: Yeah. I would, I would accept chickenpox vaccine, but hepatitis B you only tend to get if you’re on holiday, well it's, you’re much more likely to get it when you’re away

*Group 3*

Samantha: I dinnae see the point in them.
Sammy: I don’t see the point in getting a vaccine for chicken pox either.
Facilitator: How come?
Sammy: Because it's just a little rash that you’re coming out in, when you get it, and it just disappears.
Samantha: It's no very severe. You just itch when you get it.
Alison: I’d say get one for hepatitis B because that's like something serious to dae wi’ your liver.

*Group 4*

## Box 3 Girls’ understandings of, and confusion about, HPV

Facilitator: So what do you know about human papillomavirus that you get the HPV vaccine for?
Rhea: is that not like cancer, and like anyone can get cancer?
Trisha: No, is that not a gene? Like girls, like any girl could get that.
Rhea: Hang on, how do you get HPV? Because is cancer not through genes, but then…
Trisha: I think it's just like cancerous cells
Rhea: Is it?
Veronica: Yeah, it's like cancerous cells and tumours, and then you die.

*Group 11*

Deborah: Oh… it gives cervical cancer.
Wendy: Cervical cancer.
Deborah: It's sexually transmitted. You’re more likely to get it if you sleep around.
Wendy: It's a cancer in the womb. Yeah.
Deborah: You get screened for it. Women get screened for it when they’re older, and only women can get it.

*Group 12*

## Box 4 Associations drawn between measles and chickenpox

Facilitator: So what sort of things come to mind when you say measles?

Frank: Is it not kind of like chickenpox, maybe.
Brenda: A worse case, or it affects you in different places compared to chickenpox.

*Group 2*

Karl: Spots on your skin or something.
Angus: Kind of like chickenpox.

*Group 5*

Olivia: Are they not like, they’re a bit like chickenpox little spots or something?
Grace: But what are chickenpox then?

*Group 6*

Rebecca: … is that not a bit like chickenpox?
Murray: More serious though.

*Group 8*

Struan: It's related to the mumps.
Rhea: I just know ‘cats got the measles’.
Veronica: Is it like, I thought it was related to chickenpox? I thought they had some similarities.

*Group 11*

Christine: Am I right in saying you can only get measles once you’ve had chickenpox?
Wendy: I think of a bad temperature.
Deborah: Measles is more, and it lasts longer as well.
Christine: Measles is more an adult thing and chickenpox is a child thing.
Deborah: and it's more severe.
Christine: Yes.

*Group 12*
